# Surface-Induced Electronic and Vibrational Level Shifting of [Fe(py)_2_bpym(NCS)_2_] on Al(100)

**DOI:** 10.3390/ma16186150

**Published:** 2023-09-10

**Authors:** Yachao Zhang

**Affiliations:** Guizhou Provincial Key Laboratory of Computational Nano-Material Science, Guizhou Education University, Guiyang 550018, China; yachao.zhang@pku.edu.cn

**Keywords:** spin crossover, molecule–surface coupling, density functional calculations, spin state locking, critical temperature

## Abstract

It is essential that one understands how the surface degrees of freedom influence molecular spin switching to successfully integrate spin crossover (SCO) molecules into devices. This study uses density functional theory calculations to investigate how spin state energetics and molecular vibrations change in a Fe(II) SCO compound named [Fe(py)_2_bpym(NCS)_2_] when deposited on an Al(100) surface. The calculations consider an environment-dependent *U* to assess the local Coulomb correlation of 3d electrons. The results show that the adsorption configurations heavily affect the spin state splitting, which increases by 10–40 $\mathrm{kJ}\;{\mathrm{mol}}^{-1}$ on the surface, and this is detrimental to spin conversion. This effect is due to the surface binding energy variation across the spin transition. The preference for the low-spin state originates partly from the strong correlation effect. Furthermore, the surface environment constrains the vibrational entropy difference, which decreases by 8–17 $J\;{\mathrm{mol}}^{-1}\;K^{-1}$ (at 300 K) and leads to higher critical temperatures. These results suggest that the electronic energy splitting and vibrational level shifting are suitable features for characterizing the spin transition process on surfaces, and they can provide access to high-throughput screening of spin crossover devices.

## 1. Introduction

Compounds containing transition metals with six-coordinated structures and electronic configurations ranging from $d^{4}$ to $d^{7}$ have been observed to exhibit spin crossover (SCO) [1,2]. The ligands in these compounds arrange themselves in an octahedral manner, splitting the 3d orbitals at the magnetic site into two sets. These sets are the bonding $t_{2g}$ orbitals ($d_{xy},d_{yz},d_{xz}$) and the antibonding $e_{g}$ orbitals ($d_{x^{2}-y^{2}},d_{z^{2}}$). An energy gap exists between these two groups of orbitals, known as the crystal field splitting energy Δ. In certain circumstances, Δ can balance the electron pairing energy to enable both the low-spin (LS) and high-spin (HS) states to become accessible under the influence of external stimuli [3]. Due to their inherent spin bistability at the single-molecule level, these materials have potential applications in several nanoscale electronic devices, such as switches [4,5], sensors [6], and non-volatile memories [7,8]. When integrating into spintronic devices, the molecule needs to be anchored to surfaces or electrodes in a stable configuration [9,10,11]. The problem with surface-supported SCO molecules is that neither their influence on molecular functionality nor the underlying mechanism is evident at this stage. The subtle influence appears to be heavily dependent on surface environments [12,13,14,15]. In some cases, SCO molecules even suffer from spin state locking once they are deposited on solid-state substrates [16,17]. Such an issue arises partially because metallic substrates preferentially stabilize the LS state [18,19], but the electronic origin of this effect remains a puzzle in the field. A firm understanding of how molecule–surface coupling could tune SCO phenomena requires a reliable description of the materials at atomic and electronic levels [20,21].

Thus far, however, a universal approach to measuring the surface effects still needs to be developed. To this end, one must extract a series of physical features intrinsic to the spin conversion. Such features open the way to a materials genome strategy, such as the high-throughput workflow search for candidate SCO systems at interfaces. A key consideration in selecting a material feature is its availability in ground-state density functional theory (DFT) calculations. One crucial factor is the electronic energy gap (ΔE) between the HS and LS states [22,23,24]. A typical spin crossover material should have a ΔE smaller than 30 $\mathrm{kJ}\;{\mathrm{mol}}^{-1}$ [25], which is especially useful as an indicator of the possibility of a system exhibiting reversible spin transition under certain conditions. Hence, by evaluating the spin state splitting, one can efficiently figure out whether SCO properties might be preserved on a specific substrate [26,27].

There exist several difficulties, however, with the first principles calculation of ΔE. In particular, since SCO compounds are strongly correlated materials, a reliable prediction of the spin state energetics has not yet been well established within DFT calculations utilizing local or semi-local exchange–correlation (xc) functionals [28]. Therefore, it is crucial to consider the strong correlation effects and address them explicitly. One cost-effective approach to account for the localized 3d electrons present in the magnetic center involves the introduction of a Hubbard-like term, known as DFT + *U* [29,30,31,32]. In realistic materials calculations, one usually takes *U* as an empirical parameter [33,34,35,36], which is determined by fitting. The DFT + *U* method performs well compared to hybrid functionals [37,38] despite uncertainties caused by the *U* parameter, and thus, it is suitable for extended materials.

Another significant factor in the spin crossover is the vibrational degrees of freedom [39,40]. During the spin transition from a low-spin to high-spin state, some electrons move from $t_{2g}$ to $e_{g}$ orbitals. The electron excitation leads to a weakening of the metal–ligand bonds, which causes a red shift in molecular vibrational energy levels. Lowering vibrational frequencies increases entropy, the main driving force behind thermal spin crossover [41,42]. As a result, it is equally important to incorporate the phonon properties to identify spin crossover behaviors subject to the chemical environment at interfaces.

The main objective of this study is to investigate the impact of surfaces on the behavior of spin transition in terms of changes in electronic and vibrational levels. I analyzed a typical SCO molecule, [Fe(py)_2_bpym(NCS)_2_] [43]. The molecule consists of pyridine (py), 2,2${}^{\prime }$-bipyrimidine (bpym), and NCS ligands, and it was deposited on an Al(100) surface. I focused on two adsorption configurations with different molecular orientations. By conducting periodic DFT + *U* calculations, I analyzed the spin state energetics, electronic structure, interface bonding, and vibrational frequencies. This study reveals that ΔE increases in both configurations. The presence of the metal surface effectively stabilizes the LS state, resulting in a higher critical temperature or even quenching spin state switching. The increase in spin state splitting is mainly caused by the change in the adsorption energy in response to spin conversion, which the binding geometry can considerably influence. This effect can be attributed, in part, to the strong correlation of the 3d electrons. Furthermore, the calculations suggest that the entropy change across spin switching decreases due to the constraint of the surface on molecular vibrations, which further increases the critical temperature. These results imply that the influence of surface on spin crossover can be effectively quantified by the variation in energy splitting (ΔE) and in the entropy change (ΔS) derived from shifts in the vibrational density of states. These findings could help develop nanoscale spintronic devices based on workflow-oriented materials design.

## 2. Computational Details

For all calculations, I used Gpaw-20.1.0 [44], a Python-3.7.4/C electronic structure package that implements the projector-augmented wave method [45]. I set up the materials models using the Python package atomic simulation environment (ASE-3.19.0) [46]. To simulate the Al(100) surface, I utilized a slab model 6×6×3 in size, which was cleaved from a face-centered cubic crystal structure with a lattice constant of 4.05 Å. To minimize artificial interactions between periodic images, I included a vacuum layer that was 40 Å thick. I positioned the molecule 2.5 Å above the surface as an initial guess and performed local structure relaxation using an “optimize” module from ASE. As the optimizer, I used the Broyden–Fletcher–Goldfarb–Shanno (BFGS) algorithm [47], and set the force convergence criterion to 0.01 eV/Å. During the structural relaxation process, the crystal cell remained fixed while the atoms in the bottom layer of the slab model were frozen in their positions, as they would be in the bulk environment. An atom was allowed to move up to a maximum distance of 0.02 Å per iteration.

I employed the Perdew–Wang parametrization [48] of the local density approximation (LDA) as the xc functional for geometry optimization and electronic structure calculations. To account for the strong correlation effect, I adopted the LDA + *U* method [49,50,51] in the simplified rotationally invariant scheme [52]. In the LDA + *U* formulation, the parameter *U* that describes the local Coulomb correlation is redefined as $U_{\mathrm{eff}}=U-J$. For the calculations, I employed a chemical environment-dependent *U* value of 3.54 eV, which was determined via first principles methodology. This *U* value allowed for a reliable prediction of the critical temperature $T_{c}$ of the molecule under study [38].

The electronic wave functions were represented through linear combinations of atomic orbital formulations, using polarized double-ζ basis sets. Considering the size of the simulation cell, I simply included the Γ-point for the *k*-point sampling. A Fermi–Dirac smearing of occupation numbers was specified, with a width parameter ($k_{B}T$) of 0.086 eV. The DFT calculations considered spin polarization and imposed fixed spin moments of 0 and 4 $\mu_{B}$ for low-spin and high-spin states, respectively. I specified the convergence criteria for the self-consistent field (SCF) by setting the maximum electronic energy change to $10^{-6}$ eV per valence electron and the maximum integral of absolute density change to $10^{-6}$ electrons. Additionally, while examining the surface-supported molecule, I applied a dipole correction [53] along the *z*-axis.

In evaluating the critical temperature of spin crossover, one needs to consider the vibrational contributions to thermodynamic parameters such as entropy (*S*) and Gibbs free energy (*G*). The calculation was performed based on the harmonic approximation, which allowed me to obtain phonon frequencies ($\omega_{\lambda }$) and corresponding normal modes ($u_{\lambda }$). To achieve this, the mass-weighted Hessian matrix ($M^{-1/2}HM^{-1/2}$) should be diagonalized [54]. Here, M is a diagonal matrix representing atomic masses, and H is the Hessian matrix, defined as

(1) $$H_{ij}=\frac{\partial^{2}E}{\partial a_{i}\partial a_{j}}{|}_{0}=-\frac{\partial F_{j}}{\partial a_{i}}.$$

In this equation, *a* represents atomic positions, and the indices *i* and *j* run over the spatial coordinates *x*, *y*, and *z* of all dynamic atoms. The subscript 0 indicates the equilibrium geometry as a reference point. The finite difference method was used to construct the Hessian matrix H. Specifically, the forces *F* acting on each atom were computed for six displacements along the spatial directions with a displacement magnitude of Δa=0.01 Å.

## 3. Results and Discussion

### 3.1. Adsorption Configurations and Spin State Energetics

**Figure 1:**
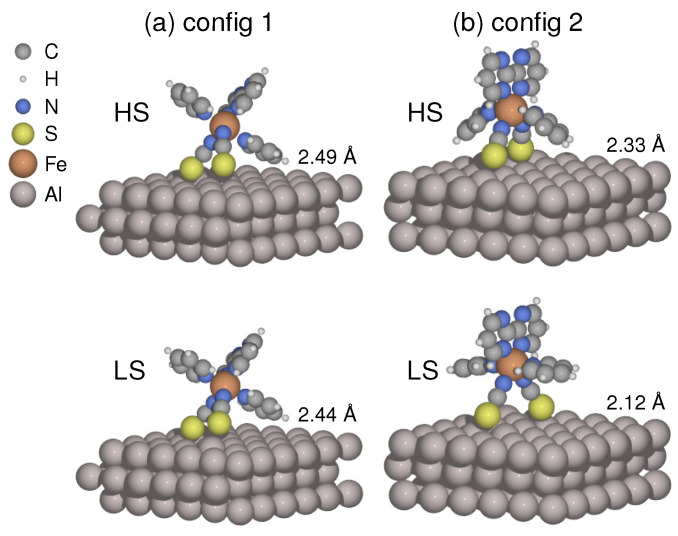
(**a**) Lying (config 1) and (**b**) standing (config 2) adsorption configurations are shown in high/low spin (HS/LS) states with average molecule–surface distances obtained by structural relaxations.

To optimize the interface structures, I focused on two types of molecular orientations of the [Fe(py)_2_bpym(NCS)_2_] molecule on the Al(100) surface. One is the lying configuration, where the two NCS groups and one of the pyridine (py) rings are in contact with the substrate (config 1). Figure 1a shows the relaxed geometries in both spin states. The other one is the standing configuration (config 2), where the two NCS groups act as main linkers, and the planar bpym ligand lies perpendicular to the surface (see Figure 1b). When the molecule undergoes LS to HS transition, the average Fe–N distance increases from 1.92 to 2.09 Å in the free molecule. The coordination sphere tends to be expanded by the molecule–surface coupling. In config 1, the metal–ligand bonds are elongated by an average of 0.01 Å and 0.02 Å for the LS and HS states, respectively. Meanwhile, the LS state remains almost unaltered regarding Fe–N distances in config 2, whereas the corresponding HS state is expanded by 0.03 Å, showing a drastic conformational change (Figure 1b).

The equilibrium molecule–surface distance is characterized by the difference between the mean height of the two sulfur atoms and that of the first-layer Al atoms in the substrate. The calculations reveal that the distance increases by 0.05 Å for config 1 and 0.21 Å for config 2 when transitioning from LS to HS states. The increase in molecule–surface separation indicates that the coupling between the molecule and the metal surface tends to be weakened in the HS state. This observation is consistent with another recent study on the SCO molecule [Fe(tzpy)_2_(NCS)_2_], which was supported by the Au(100) surface [55]. Meanwhile, the molecule–metal separation is shortened by 0.16–0.32 Å in config 2 compared to config 1, suggesting that the adsorption geometry could modulate the strength of coupling with the substrate.

**Figure 2:**
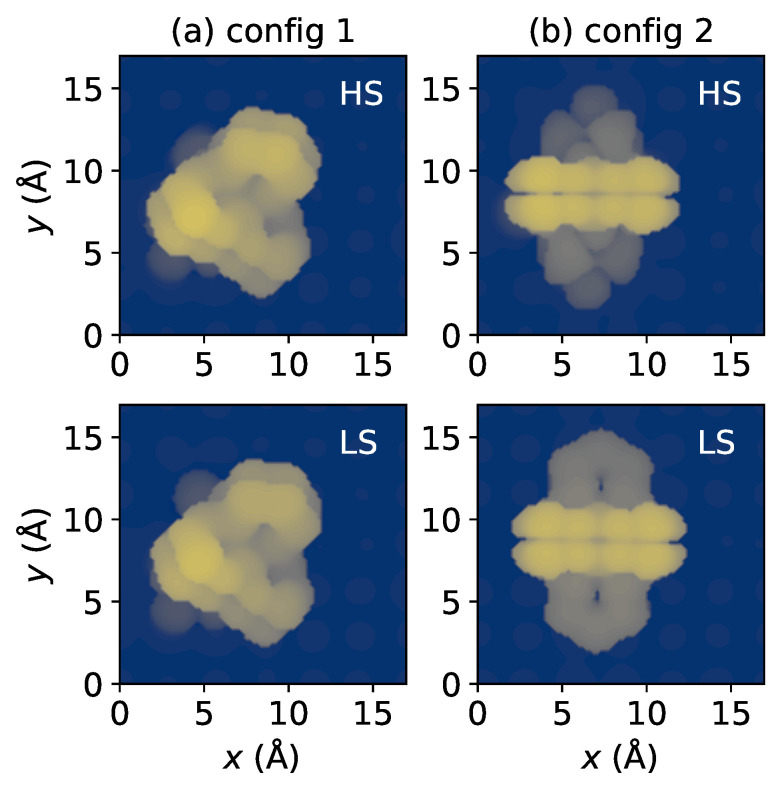
Simulated scanning tunneling microscopy (STM) images of (**a**) config 1 and (**b**) config 2 in both spin states. The computation was carried out in a constant current mode. A voltage bias of 1.5 V was applied, and the current was established at 20 nA.

Figure 2 presents scanning tunneling microscopy (STM) images simulated using the local density of states of surfaces, according to the Tersoff–Hamann method [56]. One might expect that these two adsorption styles can be easily distinguished through the STM experiment, which has been used extensively in exploring the spin state-dependent molecular conformations on metallic substrates [57,58,59]. Identifying the spin states of molecules in config 1 through STM images (Figure 2a) is difficult due to the lack of evident conformation change. For instance, the S–S distance variation is smaller than 0.1 Å. However, in the case of config 2, a noticeable difference in topographies between LS and HS states was observed (Figure 2b), indicating the unambiguous detection of spin transition for config 2 through STM studies.

The corresponding energetic properties are presented in Table 1. To show the impact of non-covalent interactions, I also conducted single-point calculations utilizing the Perdew–Burke–Ernzerhof (PBE) [60] generalized gradient approximation with dispersion corrections (PBE + D3) [61] based on the LDA + *U* geometries. Both LDA + *U* and PBE + D3 show that the surface more significantly stabilizes the LS state of config 1 than the HS state. The change in adsorption energy due to spin switching reaches more than 0.4 eV. Accordingly, the spin state splitting ΔE rises by about 40 $\mathrm{kJ}\;{\mathrm{mol}}^{-1}$ with respect to its free molecule counterpart. In contrast, the difference in adsorption energy between the two spin states is reduced to about 0.1 eV for config 2, as predicted by LDA + *U*. When transitioning from config 1 to config 2, the absolute value of the adsorption energy $E_{\mathrm{ads}}$ increases in the HS state and decreases in the LS state. Correspondingly, the ΔE of config 2 is only raised by about 10 $\mathrm{kJ}\;{\mathrm{mol}}^{-1}$ compared to that of the isolated molecule.

It is important to note that the HS state of the molecule has weaker bonding, making it more flexible than the LS state. This flexibility allows the molecule to adapt better to the surface environment. Specifically, in the standing configuration, the two pyridine rings tend to orient themselves towards the surface in the HS state, as shown in Figure 1b. This orientation enhances the anchoring of the molecule onto the substrate. This is why the ΔE of config 2 is considerably smaller than that of config 1. These results imply that the electronic tuning of spin state splitting results from the shift in molecule–surface interactions across spin conversion, which should be primarily governed by the adsorption configurations. This finding has already been demonstrated in a previous study [62], where the adsorption geometry dictates the spin transition behavior of a sandwich-type molecule supported by Cu(111). Furthermore, Table 1 indicates that config 1 is more stable in the low-spin state, while config 2 is favored in the high-spin state, suggesting an adsorption configuration change upon spin transition.

Here, including van der Waals (vdW) interactions results in greater adsorption energy in the HS state compared to the LS state for config 2. The correction gives rise to a decrease in ΔE of approximately 20 $\mathrm{kJ}\;{\mathrm{mol}}^{-1}$ relative to the free molecule case. This effect highlights the significant role of vdW interactions [63] in the renormalization of spin state splitting on surfaces. I should mention that strong molecule–substrate interactions may hinder spin switching [64,65,66], leading to the coexistence of HS and LS molecules even at low temperatures [67,68,69]. Moreover, it should be noted that although LDA fails to describe the vdW interactions physically, it reasonably captures the sizable decrease in HS–LS splitting when transforming from config 1 to config 2. Therefore, I will concentrate on the LDA + *U* findings in the subsequent discussion.

In addition, it is interesting to see whether these results could be reproduced by calculations lacking dipole correction (DC). Table 1 compares the resulting HS–LS splitting and adsorption energies delivered by the two computational setups. It shows that the adsorption energies (absolute values) are increased considerably for both spin states when DC is switched off. However, the HS–LS energy splitting is not altered significantly. This means that the effect of DC can be safely neglected if one is only interested in the electronic energy change due to spin switching.

When discussing the spin transition process from the S=0 to the S=2 state, one might be tempted to consider the intermediate spin configuration $t_{2g}^{5}e_{g}^{1}$ (S=1). However, a recent study on the [Fe(phen)_2_(NCS)_2_] compound [27] revealed that spin crossover through the S=1 intermediate state imposes a significantly higher energy barrier than direct LS→HS transition. I also performed some additional calculations on the [Fe(py)_2_bpym(NCS)_2_] compound, and found that the intermediate state is higher in energy by 0.41 eV than the HS state, and by 0.63 eV than the LS state. This indicates that the molecule is less likely to undergo spin transition through the path involving the S=1 state.

### 3.2. On-Site Coulomb Repulsion and Ligand Field

**Figure 3:**
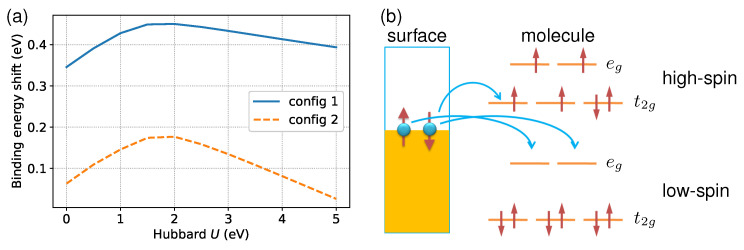
(**a**) The dependence of molecule–surface binding energy shift ($E_{\mathrm{ads},\mathrm{HS}}-E_{\mathrm{ads},\mathrm{LS}}$) on the Hubbard *U* parameter in LDA + *U* calculations, which measures the on-site Coulomb interaction. (**b**) A schematic showing how electrons transfer from the Al(100) surface to the unoccupied 3d orbitals of the molecule.

To help understand why the metallic substrate prefers LS states, I analyzed the impact of the parameter *U* on spin state energetics. As previously mentioned, the spin state splitting is highly sensitive to the shift in binding energy. Therefore, I will focus on the latter. As a function of *U*, Figure 3a shows the change in molecule–surface interactions, $\Delta E_{\mathrm{ads}}$ (i.e., $E_{\mathrm{ads},\mathrm{HS}}-E_{\mathrm{ads},\mathrm{LS}}$), for the two adsorption configurations across the spin transition. In both cases, the $\Delta E_{\mathrm{ads}}$ tends to be increased in response to the slow increment of *U* at the LDA starting point. From an electronic configuration perspective, there is a clear physical picture of this effect. Figure 3b depicts the movement of electrons from the metallic surface to the unoccupied 3d orbitals of the molecule in both spin states. Note that the LS state can accommodate electrons in both majority- and minority-spin states coming from the substrate since it has fully empty frontier orbitals. In contrast, one must overcome the Coulomb repulsion energy to inject electrons into the HS state because both $t_{2g}$ and $e_{g}$ orbitals are partially occupied. More specifically, only minority-spin electrons are accepted by the HS molecule. As a result, the coupling between the molecule and surface would be stronger in the LS state than in the HS state, in line with previous first principles findings for other SCO molecules [26,55]. Increasing *U* results in a larger $\Delta E_{\mathrm{ads}}$ due to enhanced Coulomb repulsion at the magnetic site. However, the rise in *U* above 2.0 eV produces a progressive decrement of the binding energy gap. An explanation for the phenomenon could be that, at higher *U* values, the 3d orbitals become strictly localized [70], which leads to weakened metal–ligand bonds. Consequently, the difference between HS and LS states induced by the surface reduces with an increase in *U*.

**Figure 4:**
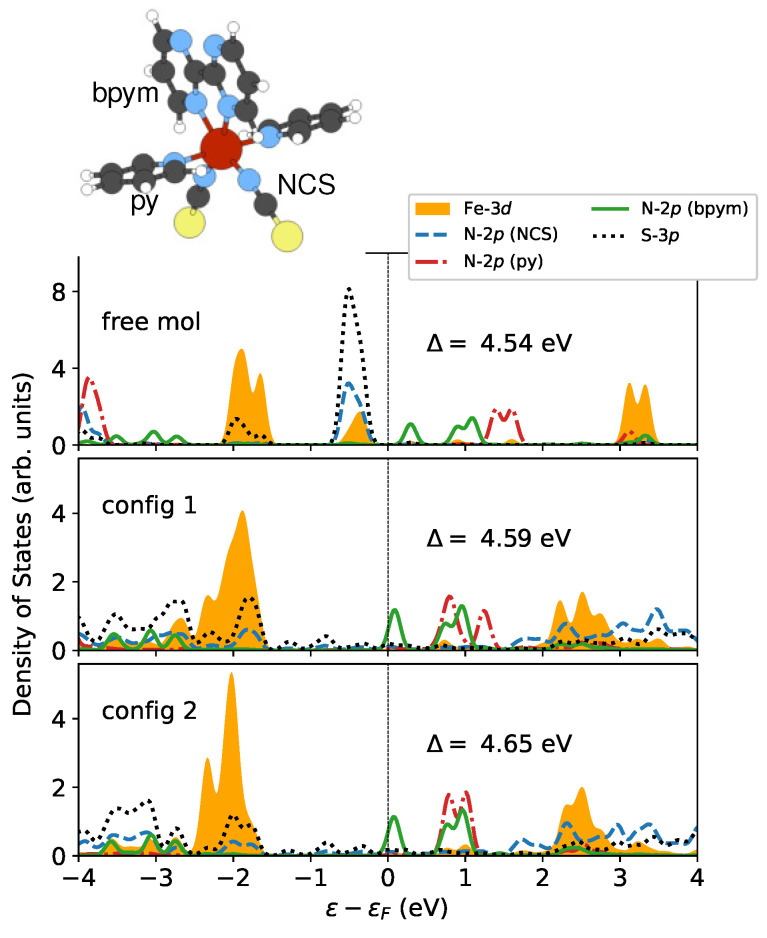
The partial density of states (PDOS) of the Fe-3d orbitals and their neighboring N-2p and S-3p orbitals in the LS state. The ligands pyridine (py), 2,2${}^{\prime }$-bipyrimidine (bpym), and NCS are labeled in the inset. Only the majority-spin components are displayed since the LS state has no spin polarization. The strength of the ligand field is characterized by the crystal field splitting Δ. This value is calculated by finding the energy difference between the centers of the occupied ($t_{2g}$) and unoccupied ($e_{g}$) Fe-3d states within the energy range of −4 to 4eV. The Fermi level $\epsilon_{F}$ is shifted to 0 eV for reference.

In order to study how the surface affects the ligand field that acts on the magnetic site, I conducted some electronic structure analysis. Figure 4 presents the partial density of states (PDOS) for the metal center (Fe-3d) and ligand orbitals, including N-2p and S-3p in the LS state. The $t_{2g}$ orbitals are fully occupied, while the $e_{g}$ orbitals are unoccupied. Under this circumstance, one can estimate the crystal field splitting Δ by analyzing the energy separation between occupied and unoccupied Fe-3d states. This gap can be calculated from relevant band centers [62]. Δ is shown to be increased by 0.05 and 0.11 eV for config 1 and config 2, respectively. The perturbations of the local coordination environment could be investigated by monitoring the evolution of $t_{2g}$ and $e_{g}$ states [71]. As illustrated in Figure 4, the rearrangement of these states is seen following the deposition on surfaces. In particular, the occupied Fe-3d orbitals exhibit a peak that moves away from the Fermi level $\epsilon_{F}$, which results in a larger crystal field splitting. This phenomenon can be attributed to the broadening of the states from the ligands, particularly the N-2p and S-3p orbitals in the NCS groups that interact directly with the surface. The increase in Δ may also contribute to the locking of the LS state. However, this contribution is too small to be notable compared to the influence of molecule–surface binding energy.

**Figure 5:**
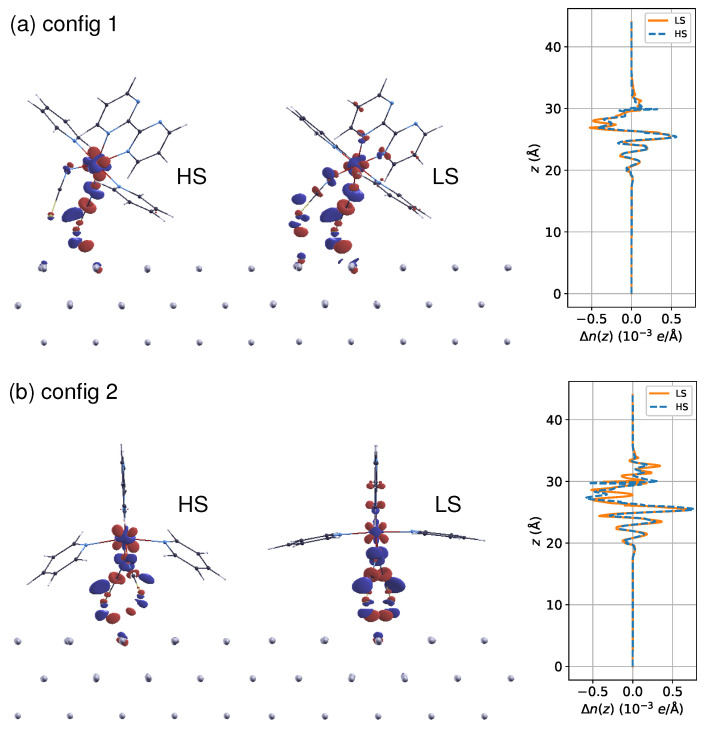
The electron density difference Δn(r) due to the interaction between the molecule and the substrate in (**a**) config 1 and (**b**) config 2 in both spin states. The integrated ones in the xy-plane Δn(z) are presented on the right. The Δn(r) is evaluated as $\Delta n\left(r\right)=n_{\mathrm{mol}/\mathrm{Al}(100)}-n_{\mathrm{mol}}-n_{\mathrm{Al}(100)}$, and $\Delta n\left(z\right)={\;\int \int \;}_{xy}\Delta n\left(r\right)dxdy$. The red and blue isosurfaces represent positive and negative Δn(r), respectively, with a cutoff of ±0.03$e/{Å}^{3}$.

The interface bonding can be inferred from the redistribution of charge density Δn(r) due to molecule–surface coupling, as displayed in Figure 5. For config 1, it appears that the LS state exhibits slightly stronger molecule–surface interactions compared to the HS state, in that both NCS ligands carry deformation charge densities (Figure 5a). In contrast, config 2 presents a much smaller difference in Δn(r) between the two spin states (Figure 5b). The reduced electronic interaction difference should explain why the binding energy shift induced by spin transition appears to be less pronounced in config 2 than in config 1. As the integrated Δn(r) within the xy-plane indicates, the molecule–surface binding strength is slightly increased for config 2, particularly in the HS state. This finding aligns well with the structural analysis mentioned above. Furthermore, it is worth mentioning that there is an apparent redistribution of electron density regarding 3d orbitals at the magnetic site in both spin states, indicating that the local spin moment could also be modified by the deposition on surfaces, as was observed in a previous work [26].

### 3.3. Spin Distribution and Charge Transfer

**Figure 6:**
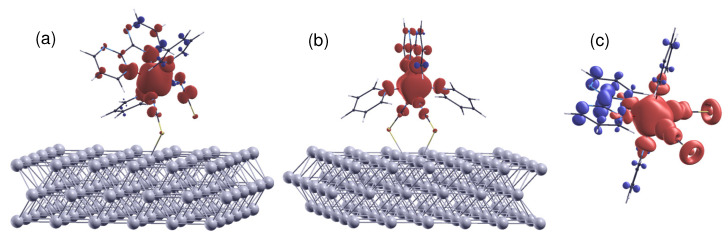
The spin density distribution ($n_{↑}-n_{↓}$) of (**a**) config 1, (**b**) config 2, and (**c**) the free molecule in the HS state. Positive and negative spin moments are represented by red and blue isosurfaces, respectively. The isosurfaces correspond to the cutoff values of ±0.002$e/{\mathrm{Bohr}}^{3}$. The delocalization of spin density at the Fe site over the ligand N atoms is revealed. Meanwhile, it can be seen that due to spin polarization, the bpym ring carries a spin moment antiparallel to that of the magnetic center. The molecule–surface coupling suppresses both effects.

To clarify the effect of molecule–surface coupling on spin distribution, I used the LDA + *U* method to compute the spin density difference $n_{↑}\left(r\right)-n_{↓}\left(r\right)$. The results reveal that both config 1 (Figure 6a) and config 2 (Figure 6b) demonstrate suppressed spin delocalization. Notably, the spin density of S atoms (Figure 6c) disappears when the molecule comes into contact with the substrate. This effect suggests that the chemical bonding leads to a quenching of the spin moments of each component. Furthermore, the spin polarization of the bpym ring carrying an antiparallel spin moment is considerably weakened. Previous studies have proposed that variations in the spin distribution of 3d complexes are caused by electron reorganization in the molecule due to charge transfer and hybridization with the substrate [72].

Using Bader charge analysis [73,74], I examined the electron transfer Δq at the molecule–metal interface and the spin moment of both the molecule and the Fe magnetic site. The results are presented in Table 2. It was observed that there is a tendency for electron transfer from the surface to the molecule. The difference in the magnitude of Δq between the LS state and the HS state is only about 0.1 *e*, but it correlates well with the strength of the molecule–surface binding that was identified from adsorption energies. Additionally, it was found that the magnetic moment of the molecule increases when it is deposited on the substrate, regardless of whether the fixed spin moment (FSM) scheme [75] is imposed. In particular, the increase in config 2 (0.12 $\mu_{B}$) is a little more obvious than that in config 1 (0.09 $\mu_{B}$) due to the relatively stronger molecule–surface interactions.

**Figure 7:**
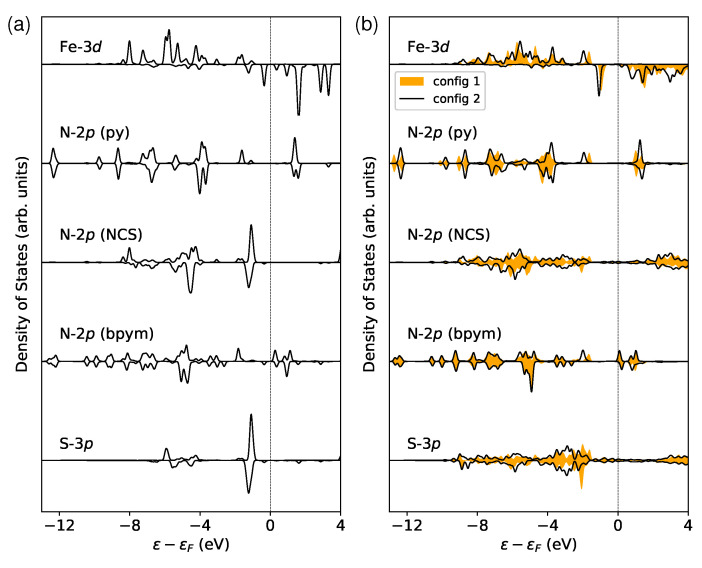
Calculated orbital- and spin-resolved PDOS of Fe-3d, N-2p, and S-3p states in (**a**) the isolated molecule and (**b**) the surface-supported molecule in the HS state. The PDOS of S-3p orbitals in the free molecule is scaled down by 1/2 with respect to other contributions. The dashed lines indicate the positions of Fermi levels $\epsilon_{F}$.

It is worth noting that the N-2p orbital in the bpym ligand, which is the lowest unoccupied molecular orbital (LUMO), belongs to the majority-spin states that are indicated by positive PDOS, as shown in Figure 7a. Therefore, if electrons are injected into the molecule, this can effectively increase the spin moment. This is in contrast to the molecule [Fe(phen)_2_(NCS)_2_], where the LUMO is a minority-spin state. Consequently, electron accumulation tends to reduce the molecular magnetic moment [27]. Additionally, it was observed that the local magnetic moment of Fe is reduced by 0.09 $\mu_{B}$ after being deposited, which is similar to the predictions for the [Fe(phen)_2_(NCS)_2_] molecule supported by the same substrate [27]. Since all empty orbitals are purely minority-spin states at the high-spin Fe site (Figure 7b), the electron supply to the magnetic center would always lead to a reduction in the local spin moment. Moreover, it should be noted that the S-3p states are broadened considerably and shifted toward lower energy in both cases (Figure 7b). As a result, the spin polarization is largely suppressed at the interface.

### 3.4. Work Function and Surface Dipole

**Figure 8:**
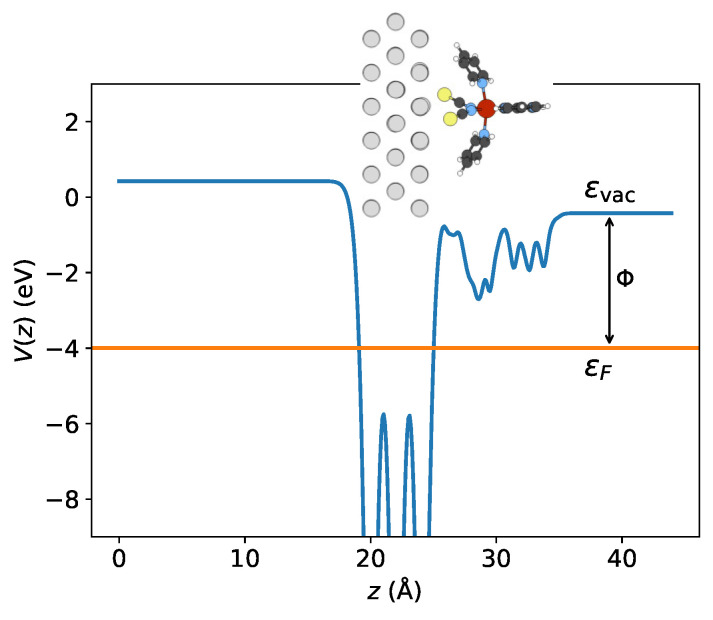
The xy-averaged electrostatic potential of config 2 in the HS state calculated by LDA + *U*. The potential, V(z), is defined as the average value of V(x,y,z) over the surface area (*S*) of the simulation cell within the xy-plane, $V\left(z\right)=\frac{1}{S}{\;\int \int \;}_{S}V\left(x,y,z\right)dxdy$. The work function Φ can be evaluated from the splitting between vacuum level $\epsilon_{\mathrm{vac}}$ and the Fermi level $\epsilon_{F}$.

Then, I attempted to explore the charge transfer phenomenon by analyzing the work function. Table 3 presents the evaluated vacuum levels $\epsilon_{\mathrm{vac}}$, Fermi energies $\epsilon_{F}$, and the resulting work functions Φ for both configurations. Due to the interface electric dipole, the Fermi energy is shifted upward relative to the vacuum level, such that the work function is lowered (see Figure 8). It has been identified that the alteration in work function is directly proportional to the electric dipole moment along the *z*-axis, $\mu_{z}$ [26]. Comparing both adsorption structures, it is evident from Table 3 that the surface dipole $\mu_{z}$ is slightly larger in the low-spin state than in the high-spin state. Since the electronic transformation creates an accompanying structural modification, the decrease in dipole moment is mainly caused by the molecular expansion upon LS to HS transition. Thus, the LS state experiences a more pronounced reduction in work function, as observed in the [Fe(phen)_2_(NCS)_2_] compound deposited on both magnetic and non-magnetic substrates [26]. This suggests that the LS state may receive more electrons due to the decrease in work function, which makes electron transfer at the interface easier. These findings are in line with the Bader analysis results presented in Table 2.

### 3.5. Vibrational Contributions

**Figure 9:**
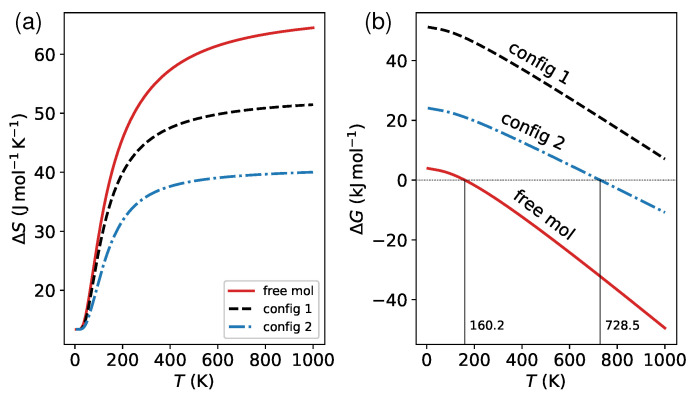
(**a**) The temperature-dependent difference in entropy, ΔS(T), resulting from changes in the spin degeneracy and accessible vibrational levels caused by the spin transition. (**b**) The Gibbs free energy change calculated using the formula ΔG(T)=ΔH(T)−TΔS(T). The enthalpy change, ΔH, consists of three components: spin state splitting, zero-point energy correction, and finite-temperature vibrational energy. Using a secant algorithm, the critical temperature, $T_{c}$, can be determined by finding the numerical root of the equation ΔG(T)=0.

Finally, I explored the impact of surface on the vibrational contributions to spin transition. Following previous work [38], I performed a thermodynamic analysis of the phonon frequencies (ω) to determine the entropy change (ΔS) and variation in Gibbs free energy (ΔG) induced by the spin transition. The results are presented in Figure 9. Note that the ΔS varies sharply with respect to the temperature *T* below 200 K, and reaches saturation when *T* is above 400 K (see Figure 9a). For this reason, at high temperatures, the ΔG varies linearly with *T*, as shown in Figure 9b. That is why, in many cases, the critical temperature $T_{c}$ can be estimated simply by ΔH/ΔS, where ΔH represents the enthalpy change. Previous studies [76,77] have found that molecular vibrations in low-frequency regimes (soft modes with frequencies below 600 ${\mathrm{cm}}^{-1}$) play a significant role in determining ΔS. At a temperature of 300 K, the change in entropy calculated based on these modes accounts for over 90% of that based on the entire spectrum for both isolated and adsorbed molecules. This implies that the primary driving force for spin crossover arises from normal modes associated with ligand bending and deformations of the coordination sphere. These patterns are affected strongly by the molecule–surface coupling. As such, the decrease in ΔS on surfaces (Figure 9a) can be attributed to the constraint of the surface environment. The results of the calculations indicate that the lying (config 1) and standing (config 2) configurations have reduced the ΔS by 8 and 17 $J\;{\mathrm{mol}}^{-1}\;K^{-1}$, respectively, at 300 K. This ΔS reduction results in an upward shift of 568 K in $T_{c}$ for config 2 compared to the free molecule (Figure 9b). However, it should be noted that the increase in $T_{c}$ should also be ascribed in part to the slight rise in the HS–LS splitting ΔE (see Table 1). The much higher $T_{c}$ (1137 K) in config 1 is mainly due to its significantly larger ΔE than the isolated molecule.

**Figure 10:**
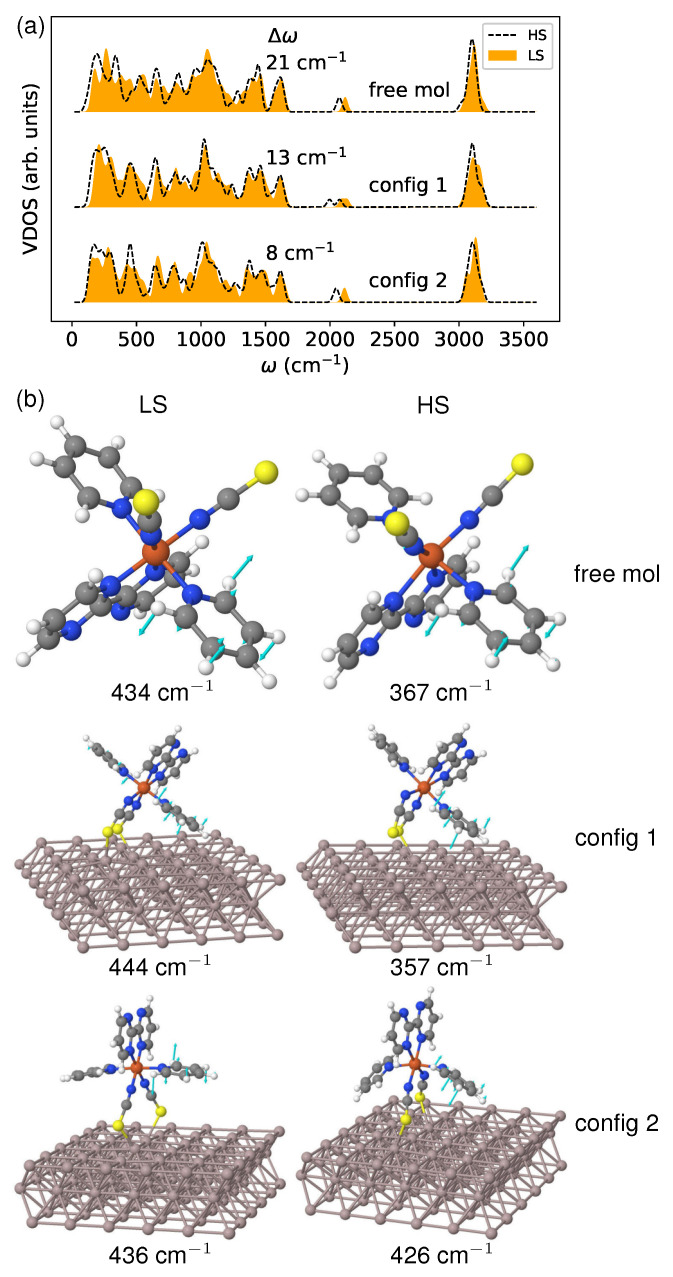
(**a**) The vibrational density of states (VDOS) of the molecule in both spin states. The discrete phonon frequencies were smeared using Gaussian broadening with a width parameter of 20 ${\mathrm{cm}}^{-1}$ to generate the spectrum. The red shift (Δω), measured in ${\mathrm{cm}}^{-1}$, is shown in the figure, and was determined by calculating the separation between the HS and LS phonon band centers. (**b**) The comparison of vibrational modes associated with out-of-plane bending (boating) of a pyridine ligand between the free molecule and the one in contact with the metallic substrate.

In Figure 10a, I present the calculated vibrational density of states (VDOS). It is evident from the VDOS that there is an appreciable shift towards lower frequencies due to the LS→HS transition. This shift occurs because the transfer of electrons from the bonding $t_{2g}$ to the antibonding $e_{g}$ orbitals weakens the metal–ligand bonds. The red shift magnitude (represented by Δω) is 13 and 8 cm${}^{-1}$ for config 1 and config 2, respectively. This result agrees with the entropy change (ΔS) obtained from thermodynamic analysis and illustrated in Figure 9a. The free molecule demonstrates an even more significant shift (21 cm${}^{-1}$), leading to a greater entropy difference than both deposited molecules. This suggests that vibrational level shifting can accurately characterize the vibrational contributions to spin crossover, even when strong coupling exists between molecules and substrate.

To demonstrate the influence of the surface environment on vibrational frequencies, I analyzed the boating mode of the pyridine ligand in the low-frequency range, as depicted in Figure 10b. Regarding config 1, the molecular configuration remains mostly unchanged after deposition in both spin states. As a result, there is only a slight increase in the mode frequency of 10 ${\mathrm{cm}}^{-1}$ in the LS state, and a decrease of the same amount in the HS state, compared to the free molecule. However, for config 2, the HS molecule undergoes noticeable deformation, making it more susceptible to the surface environment than the LS molecule. In the LS state, there is only a minor frequency shift of 2 cm${}^{-1}$, while in the HS state, there is a significant increase in the phonon frequency of 59 cm${}^{-1}$ due to conformational changes. The observed effect can be attributed to a substantial alteration in the bonding feature during spin transformation. This highlights the crucial role that surface degrees of freedom play in modulating the vibrational contributions to the spin crossover.

## 4. Conclusions

In summary, I conducted DFT calculations to study the spin crossover molecular material [Fe(py)_2_bpym(NCS)_2_] on the Al(100) surface. The molecule was deposited in both lying and standing adsorption configurations. The findings indicate that the spin state energetics and vibrational frequencies are sensitive to the interface structure. The significant electronic tuning of HS–LS splitting is mainly caused by the strong coupling at the interface, which is detrimental to spin switching. A detailed analysis of the electronic structure, bonding, and charge transfer revealed that the stabilization of the low-spin state originates from the shift in the binding energy upon the spin conversion. This phenomenon can be partly attributed to the strong correlation effect of the 3d electrons. Considering the importance of molecule–surface coupling, it is crucial to effectively consider the van der Waals interactions on top of the strong correlation effect. More work needs to be performed to address this problem. The results of the thermodynamic analysis show that the entropy increase across spin transition is considerably lowered due to the surface constraint. Consequently, this could shift the critical temperature $T_{c}$ towards higher values. The experimentally observed spin state locking on metallic substrates could be explained by the dramatically increased $T_{c}$ to over 500 K. Additionally, one can expect spin state splitting and vibrational level shifting to be applied in a materials genome approach to accelerate the discovery of surface-supported spin crossover systems.

## Figures and Tables

**Table 1:** The adsorption energies ($E_{\mathrm{ads}}$) in both high-spin (HS) and low-spin (LS) states, as well as HS–LS splitting ΔE for the two adsorption configurations, calculated with (w/) and without (w/o) dipole correction (DC). The ΔE for the free molecule was predicted to be 20.8 and 63.8 $\mathrm{kJ}\;{\mathrm{mol}}^{-1}$ by LDA + *U* and PBE + D3, respectively.

	Config 1		Config 2	
	**w/ DC**	**w/o DC**	**w/ DC**	**w/o DC**
LDA + *U*				
$E_{\mathrm{ads},\mathrm{HS}}$ (eV) ^1^	−0.86	−1.82	−1.00	−1.95
$E_{\mathrm{ads},\mathrm{LS}}$ (eV)	−1.29	−2.30	−1.10	−2.07
ΔE ($\mathrm{kJ}\;{\mathrm{mol}}^{-1}$)	61.5	67.1	31.0	31.7
PBE + D3				
$E_{\mathrm{ads},\mathrm{HS}}$ (eV)	−0.98	−1.81	−1.31	−2.16
$E_{\mathrm{ads},\mathrm{LS}}$ (eV)	−1.39	−2.23	−1.09	−1.93
ΔE ($\mathrm{kJ}\;{\mathrm{mol}}^{-1}$)	104.0	104.2	42.8	42.2

^1^$E_{\mathrm{ads}}=E_{\mathrm{mol}/\mathrm{Al}(100)}-E_{\mathrm{mol}}-E_{\mathrm{Al}(100)}$.

**Table 2:** The magnitude of electron transfer (Δq) from the substrate to the molecule, the molecular magnetic moment (*m*), and the local spin moment at the Fe site ($m_{\mathrm{Fe}}$) in the high-spin state obtained according to Bader analysis.

	Config 1	Config 2	Free mol
$\Delta q_{\mathrm{HS}}$ ^1^	0.42	0.44	
$\Delta q_{\mathrm{LS}}$	0.53	0.54	
*m* ($\mu_{B}$)	4.09 (4.10) ^2^	4.12 (4.14)	4.00 (4.05)
$m_{\mathrm{Fe}}$ ($\mu_{B}$)	3.77 (3.77)	3.77 (3.77)	3.86 (3.86)

^1^$\Delta q=q_{\mathrm{adsmol}}-q_{\mathrm{freemol}}$; ^2^ Results obtained without constraint on total spin moment are shown in parentheses.

**Table 3:** Calculated vacuum level $\epsilon_{\mathrm{vac}}$ on the top side, Fermi level $\epsilon_{F}$, work function Φ, and surface dipole moment $\mu_{z}$ for HS and LS states.

	Config 1		Config 2	
	**HS**	**LS**	**HS**	**LS**
$\epsilon_{\mathrm{vac}}$ (eV)	−0.53	−0.55	−0.42	−0.49
$\epsilon_{F}$ (eV)	−3.88	−3.87	−3.99	−3.93
Φ (eV)	3.35	3.32	3.57	3.44
$\mu_{z}$ (*e* Å)	1.74	1.80	1.38	1.60

## Data Availability

The data that support the findings of this study are available from the corresponding author upon reasonable request.
